# Applications of Tea (*Camellia sinensis*) and Its Active Constituents in Cosmetics

**DOI:** 10.3390/molecules24234277

**Published:** 2019-11-24

**Authors:** Wojciech Koch, Justyna Zagórska, Zbigniew Marzec, Wirginia Kukula-Koch

**Affiliations:** 1 Chair and Department of Food and Nutrition, Medical University of Lublin, 4a, Chodźki str., 20-093 Lublin, Poland; zbigniew.marzec@umlub.pl; 2 Chair and Department of Pharmacognosy, Medical University of Lublin, 1 Chodźki str., 20-093 Lublin, Poland; zagorska.justyna@gmail.com (J.Z.); virginia.kukula@gmail.com (W.K.-K.)

**Keywords:** tea plant, skin care cosmetics, dermatology, *Camellia sinensis* (L.) Kuntze, catechins, Theaceae

## Abstract

Studies on the cosmetic applications of plant extracts are increasingly appearing in the scientific literature, which is due to the growing popularity of skincare products around the world. In the light of the observed changes, a return to natural treatment and skincare with cosmetics free of harmful substances or toxic preservatives is visible. Currently, tea extracts, due to their rich composition and various biological actions, play an important role among the dietary supplements and cosmetics. This review is intended to collect the reports on the properties of the tea plant, its extracts and preparations in cosmetology: for skin care products and for the treatment of selected dermatological diseases. Particular attention is paid to its antioxidant, anti-hyaluronidase, anti-inflammatory, slimming, hair-strengthening, photoprotective and sealing blood vessels properties.

## 1. Introduction

Cosmetics market continues to grow globally within the last decade. The growth of the upper middle class, an increasing number of senior citizens around the world and the expansion of online beauty spending and social networks, which certainly set new trends among the consumers, are all having an impact on an increasing interest in skin care products. Natural products as the cosmetics’ ingredients are often associated with safety, marked activity and good quality. That is why a great interest in cosmetic products of natural origin can be observed [1].

Tea plant itself and its extracts together with their centuries-old tradition of use play an important role on the cosmetics market. In general, cosmetics products containing tea extracts rich in polyphenols have a positive effect on the skin appearance and ameliorate skin damage, erythema and lipid peroxidation following UV exposure [2]. An increasing number of cosmetics containing tea extracts, especially those produced using green tea infusions, but recently also black and white teas, encourage the authors to provide a review, that is focused on the application of tea in cosmetics. For the moment only a few review publications undertake the topic, however, the majority of them were published more than a decade ago. Due to this fact the authors found it necessary to prepare a more up-to-date manuscript that includes the information that have been spread in recent years, in the times of a constant and significant growth of the cosmetic industry [2,3,4]. Some of the above mentioned previously published references were also focused on the treatment of specific skin diseases only and represent rather dermatology than cosmetic applications [5,6], or they described the activity of specific tea ingredients, e.g., caffeine [7] than the extracts per se. Therefore the aim of the present review is to collect the reports on the properties of the tea plant, its extracts and preparations in cosmetology: for skin care products and for the treatment of selected dermatological diseases.

## 2. The Chemical Composition of Tea Plant (*Camellia sinensis* (L.) Kuntze)

Tea plant is a rich source of bioactive components. According to the scientific literature it contains almost 4000 metabolites, among which the group of polyphenols constitutes a more than one-third share [8]. Tea infusions deliver approx. 2–3% flavonol glycosides (kaempferol, myricetin and quercetin), whereas their aglycones most often remain in the plant matrix upon water extraction due to their lower polarity. The flavanols present in tea infusions, also called catechins, constitute as much as 20–30% of tea’s dry matter. They are responsible for its taste: bitterness and astringency [9]. The composition of tea varies depending on the fermentation process applied. Black tea contains (−)-epigallocatechin gallate (EGCG), (−)-gallocatechin gallate (GCG), (−)-gallocatechin (GC), (+)-catechin (C), (−)-epicatechin (EC), gallate (−)-epicatechin (ECG) and (−)-epigallocatechin (EGC) [10,11,12] in contrast to green tea that is rich in EGCG—present in the highest concentration, ECG, EC and EGC [13]. The differences in the composition of these two the most common types of tea is strictly related to the production process. To obtain green tea freshly harvested leaves of *Camellia sinensis* are treated with hot steam to prevent fermentation and are later subjected to drying. On the other hand, black tea is produced from the leaves, which are first dried, then rolled, ground and finally fermented. This is the fermentation process that induces the oxidation of polyphenols triggered by the influence of polyphenol oxidases [14]. This process results in the transformation of simple flavonoids (e.g., catechins) into more complex structures, like thearubigins (TR), theaflavins (TF) and theobrownins (TB) [10,11,12].

Therefore, the catechins’ concentration is inversely proportional to the degree of leaf processing [15]. Their highest content was noted in green tea, then in oolong tea and in the end-in black tea, which is due to a strong fermentation process that the latter type of tea is subjected to [10,16,17]. On the other hand black tea is a rich source of TR and TF [16] and oolong tea-of theasinensins and other condensed phenolic compounds [18]. TR, whose molecular weight is from 700 to 40,000 Da, gives the brew of black tea a taste and a reddish-black color [19]. In contrast, theaflavins are responsible for a golden yellow color of the infusion [9]. Catechins are certainly the best studied compounds of plant origin. However, still not much is known about the chemical structures and pharmacological properties of the catechins’ conjugates, as they are difficult to identify and isolate from tea leaves. Some of the theaflavins have been, however, identified in tea extracts and include: 3-3′-theaflavin digalusate (TF3), theaflavin 3-gallate (TF2B), theaflavin 3-gallate (TF2A) and theaflavin (TF1) [20]. 

**Figure 1:**
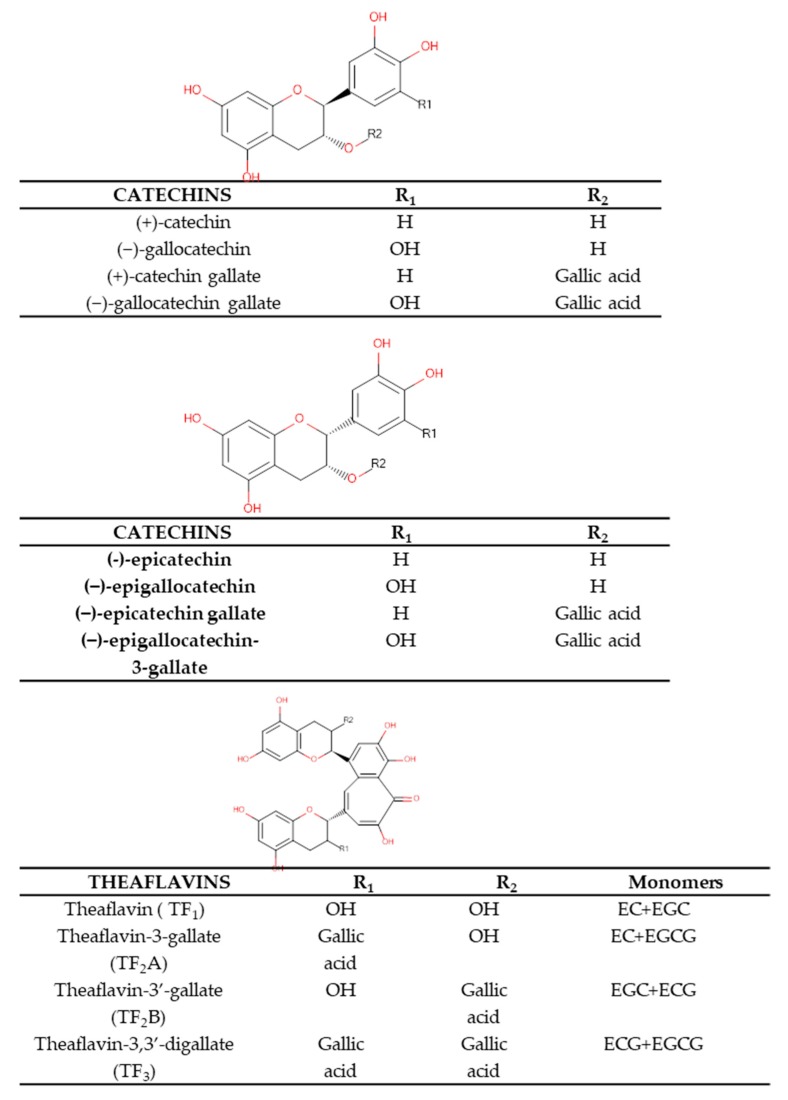
Chemical structures of the major secondary metabolites present in tea leaves.

Different types of tea are also rich sources of simple phenolic compounds (gallic acid—GA, *p*-coumaric acid and caffeic acid) and their derivatives: theogaline and chlorogenic acid [21,22,23,24]. Other compounds present in tea include purine alkaloids (theophylline, theobromine and caffeine (theine)), amino acids, theanine [25], carbohydrates, lipids (linoleic and linolenic acids), volatile compounds, pigments (carotenoids and chlorophylls), vitamins (A, C, E, K and B) and chlorophylls [26]. It also contains numerous mineral elements such as iron, zinc, sodium, magnesium, chromium, phosphorus, potassium, titanium, manganese, nickel, copper, aluminum, silver and bromine [23,27,28,29,30,31]. The structures of tea main catechins and theaflavins were presented in Figure 1. 

## 3. The Application of Tea Extracts in Cosmetics

**Figure 2:**
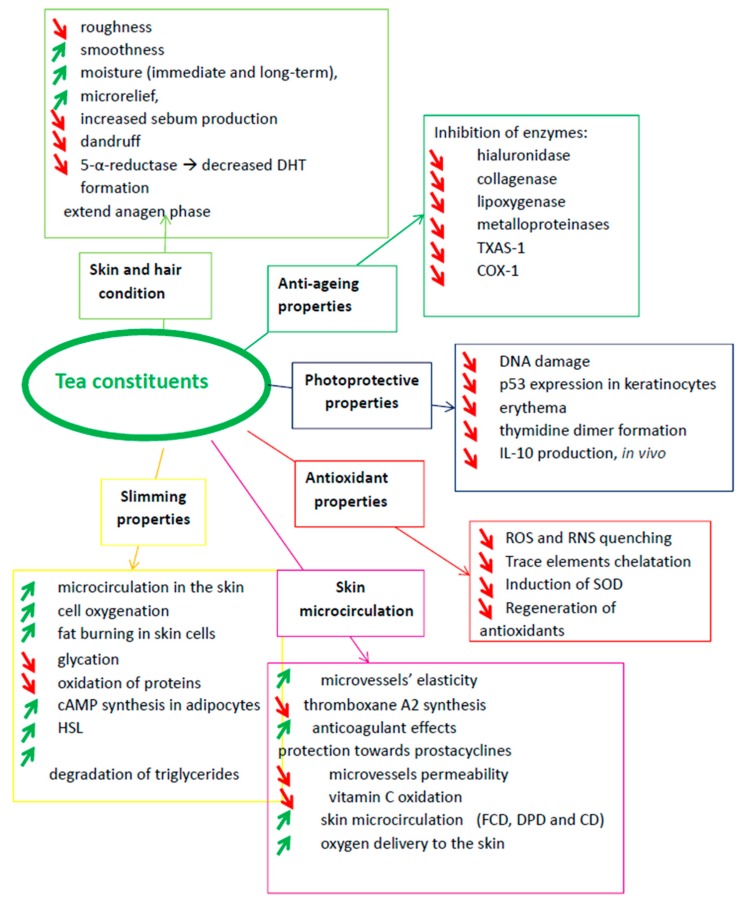
The major cosmetic properties of catechins (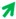
—stimulation, 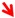
—inhibition).

Tea extracts possess a wide spectrum of biological activities, which makes them precious components not only for pharmaceutical applications, but also for cosmetics industry. Among these activities antioxidant, photoprotective, anticellulite, slimming, improving skin, hair and microcirculation condition properties should be underlined (Figure 2). They will be further reviewed and discussed. 

### 3.1. The Application of Tea Plant in Cosmetics and its Activity for the Skin Biochemistry

The skin is the largest organ of the human body, which accounts for approximately 15% of its weight. It is composed of three layers: the epidermis, the dermis and subcutaneous tissue [32]. The epidermis is composed mainly from two types of the cells: keratinocytes and dendritic cells. The morphology and position of the keratinocytes condition the structure of epidermis into four layers: stratum germinativum (horny cells, including the basal cell layer), stratum spinosum (the squamous cell layer), stratum granulosum (the granular cell layer) and stratum corneum (cornified or horny cell layer) [33,34,35]. The epidermis is a constantly renewing layer and gives rise to derivative structures, such as pilosebaceous apparatuses, nails and sweat glands. Cells from the stratum germinativum undergo proliferation cycles and continuously provide new cells for the outer epidermis. The epidermis is a dynamic layer, in which the cells are in a constant unsynchronized motion, constantly differentiating into different types of cells [35]. Each layer of the skin possesses different functions and is differently supplied with nutrients. The effects of the tea plant and its active constituents differ depending on the skin layer. In the stratum corneum, this effect is mostly due to the strong antioxidant activity of tea extracts. In deeper layers of the skin, the tea polyphenols exhibit significant protective effects against the ultraviolet radiation and affect the activity of various enzymes. By the inhibition of lipoxygenase, metalloproteinase, hyaluronidase and collagenase, tea and its extracts significantly delay the signs of skin aging. This is because these enzymes have a destructive effect on cellular cement lipids, such as hyaluronic acid, elastin and collagen, which are important components for the skin [35]. 

When administered on the dermis, these compounds improve microcirculation and the condition of blood vessels, which results in a better skin nutrition and oxygenation. Moreover, polyphenols have a protective effect towards vitamin C, because they prevent its oxidation. This is an important issue from a cosmetic point of view, as vitamin C is involved in the synthesis of collagen, a main protein of blood vessels and skin.

Tea polyphenols also affect the sealing of blood vessels by protecting hyaluronic acid and inhibiting histamine release. Their influence on the activity of adrenaline, which contracts blood vessels, is also important. Low levels of this hormone lead to weakening of the vessels, because their contractions are then short, but frequent. The polyphenolic compounds from tea plant indirectly strengthen blood vessels, as they prevent the oxidation of adrenaline. In addition, they improve blood flow through the inhibition of platelet aggregation by different mechanisms, e.g., by hindering the thrombin proteolytic activity and inhibiting the tyrosine kinase Syk and Lyn activities [36]. According to Lee and co-investigators EGCG, the major green tea catechin, significantly inhibits cyclooxygenase (COX)-1 and thromboxane synthase (TXAS) production in platelets, which are two major enzymes responsible for the platelet aggregation. It is worth mentioning that, in the case of EGCG, this effect was even stronger as compared to a nonsteroid anti-inflammatory drug aspirin, which is commonly used to inhibit thrombotic disease-associated platelet aggregation [37]. 

Tea polyphenols have anti-inflammatory activity, which significantly improves skin microcirculation. This particular action is related to the radical scavenging properties of tea polyphenols, which decelerate the inflammatory processes, which disturb microcirculation and protect the intracellular cement lipids. Moreover, an inhibition of nitric oxides, prostaglandins, thromboxanes and leukotrienes, which are the main mediators of inflammation have been observed upon the administration of tea extracts [37,38,39].

### 3.2. Antioxidant Activity

Antioxidant properties of polyphenolic compounds are widely known and were described in numerous scientific papers [5,40,41,42]. Due to their specific structure, the antioxidant activity of polyphenols may be related to almost all phenolics, however, it was proved that aglycones are stronger antioxidants, compared to their corresponding glycosides [43]. All tea catechins were described as very strong antioxidant agents based on in vitro [44,45] and in vivo studies [46,47,48,49]. Table 1 shows some selected results on antioxidant properties of tea extracts in relation to other extracts used in cosmetics. A precise direct comparison of different antioxidant studies is often difficult due to a variety of antioxidant tests applied and various methodological protocols used, however, some tendencies can be observed. Taking into account that, except water, tea is the most widely consumed beverage in the world, tea catechins are considered as the most important antioxidant substances present in human diet [5,21].

The effect of tea catechins towards the free radicals is multidirectional and includes:

- Direct quenching of reactive oxygen oxygen (ROS) and nitrogen (RNS) reactive species;
- Chelatation of trace elements that are involved in free radicals generation (e.g., copper or iron);
- Enhancement of endogenic antioxidant enzymes production (SOD (superoxide dismutase) and glutathione);
- Inhibition of enzymes involved in ROS generation (glutathione S-transferase, microsomal monooxygenase, mitochondrial succinoxidase or NADH oxidase);
- Protection and regeneration of antioxidant compounds (vitamin C or E) [5,47,48].

Antioxidant properties of tea are the major and the oldest application for that cosmetics industry needs *Camellia sinensis* extracts. Since the discovery of free radicals theory it became obvious that free radicals can trigger chain reactions, which cause damage of biological macromolecules like proteins, lipids and nucleic acids, and as a result are very harmful to skin and mostly responsible for skin aging. Since oxidative stress is one of the most important processes determining skin ageing, natural antioxidants are considered to be the most important factors in the prevention of this process [56]. Polyphenols are able to protect and restore the content of vitamin C, which is an important co-enzyme in the production of collagen—an important protein responsible for the skin elasticity and strength, building up its structure along with elastin and keratin [34]. Different types of tea, including black, white and green, have been used to produce skin care formulations, however, the latter one due to the highest concentration of polyphenols (EGCG and EGC) can be found the most often in cosmetics formulations. Other ingredients of tea plant, like previously described vitamins, lipids and pigments could also provide additional benefits to the skin, like moisturizing and protective effects [4,57,58,59]. Some selected trials on the antioxidant properties of tea plant leaf extracts have been collected in the Table 2.

### 3.3. Photoprotective Activity

UV radiation (UVR; 280–400 nm) may be very detrimental to the skin. There are proofs, which indicate that UVR induces skin carcinogenesis by multiple mechanisms, including direct DNA damage and indirect caused by ROS. UVR also induces cutaneous immunosuppression, potentially allowing dysplastic cells to go undetected and progress to neoplasms [60,61,62]. The genotoxic and cytotoxic effects of UVR in skin are well documented, and as a result malignant as well as non-melanoma skin cancers occur. In the UK there are over 40,000 of a new cases of skin cancers each year, of which 10% are malignant melanomas, with a significant risk of mortality [63]. Therefore there is a great need for searching effective photoprotective agents, with low side effects, and natural products may be very helpful to find such.

Tea leaf extract absorbs ultraviolet radiation, due to which it protects the skin against its harmful effects. It is a component of photoprotective cosmetics for daily care [64,65]. Additionally polyphenolic compounds present in tea have strong antioxidant activity (which was reviewed above) and due to it scavenging different free radicals, which are produced during UV radiation. Tea extracts and the active compounds that they contain are increasingly applied in sunscreen cosmetics. EGCG prevents the negative effects of UV radiation on the skin, through the inhibition of collagenases, anti-inflammatory and anti-cancer properties towards the skin cells. Recent findings revealed that green tea extract should be combined with zinc oxide and titanium dioxide in sunscreen preparations. This combination increases skin protection against damage caused by UVA and UVB rays. Tea extract, alone, protects only against UVB radiation [66,67]. 

Recently several studies confirmed that tea and its active constituents, when taken orally or applied topically, effectively protect skin against UVR. Morley and co-investigators proved that consumption of 540 mL of green tea infusion significantly inhibited UVR-induced damage to peripheral blood cells. Moreover obtained in vivo data were confirmed in in vitro experiments, in which EGCG was proved to dose-dependently reduce UVR induced cells DNA damage in fibroblasts and keratinocytes cell cultures [63]. Mnich and co-investigators revealed that topical administration of a treatment lotion containing 4% green tea extract to skin patches irradiated with UVB (up to 100 mJ/m^2^) significantly reduced UV-induced p53 expression in keratinocytes. The number of apoptotic keratinocytes (sunburn cells and TUNEL-positive cells-Terminal transferase dUTP-nick-end labeling assay) was also significantly decreased. On the other hand UV-induced erythema and thymidine dimer formation were also significantly affected. The authors concluded that topical application of a lotion rich in green tea extract even at low, cosmetically usable dosages efficiently reduces UVB-mediated epithelial deterioration and therefore green tea extract should be considered as suitable everyday photochemopreventive agents [68]. Not only green tea but also white tea is considered as an effective photoprotective agent. Camouse and co-workers investigated skin samples obtained from volunteers or skin explants treated with white or green tea after UV irradiation. Tea extracts were administrated topically in a special cosmetic vehicle containing deionized water, 1,3 butylene glycol, carbopol 980 triethanolamine and methyl paraben. A study was a double-blind placebo-controlled assay. The experiment revealed that application of white and green tea to a UV-irradiated skin caused a 22% and 35% reduction in CD1a+ (epidermal Langerhans cells) staining relative to unirradiated skin, respectively. Moreover, application of both teas significantly reduced oxidative DNA damage caused by UVR. As both agents were characterized by SPF 1, it was concluded that their photoprotective activity towards the skin’s immune system was not connected with their direct UV absorption or a “sunscreen” effect. Additionally white tea, because of its lighter color, was considered more acceptable to use in topical preparations, especially for a regular use around the face [62].

Photoprotective effects of green tea may be strengthened when using in combination with another herbal extract. The market contains a large number of skin care formulations containing different mixtures of botanical extracts with the claim that the combination provides enhanced skin effects. However, so far few studies have confirmed their topical effect. One of the plant species studied in a combination with green tea in a cosmetic formulation was *Ginkgo biloba*, which was proved to significantly increase photoprotective activity of green tea extract. Topical formulation containing 6% w/w of each extract (green tea and *Ginkgo biloba* glycolic extracts) was applied to a dorsal skin of 24 male albino hairless mice (HRS/J-hairless, Jackson, Bar Harbor, ME, USA) in two different areas with 5 mg/cm², 15 min prior to UVA/UVB irradiation. The study revealed that combination of both extracts significantly decreased skin damage (dryness, irritation, presence of erythema, sunburn cell formation and epidermal hyperplasia) caused by UVR. This effect was much stronger in comparison to the application of green tea and *Ginkgo biloba* separately. Photoprotective activity of both herbal extracts was not due to their UV-absorption ability, but to biological effects caused in the skin, which were much stronger when both extracts were administrated together [69]. 

In general, different animal models and in vitro studies confirmed that topical treatment with green tea polyphenols reduced UVB-induced inflammatory responses, immunosuppression and oxidative stress, which may led to skin carcinogenesis (Table 2). It was also proved that external application of EGCG decreased immunosuppressive interleukin (IL)-10 production at UV irradiated skin and draining lymph nodes. Moreover, studies performed on animals revealed that topical application of green tea catechins prior to exposure to UVB protects not only against local but also systemic UVR-induced immune suppression [70]. 

### 3.4. Anti-Cellulite and Slimming Properties 

Cellulite (gynoid lipodystrophy), often called ‘orange peel effect’, is a typical women’s ailment, which mainly appears on the thighs and buttocks [71]. It is a complex disorder in which microcirculatory and lymphatic systems as well as extracellular matrix are involved. Increased lipolysis can be observed, which lead to increased production of diglycerides, monoglycerides, free fatty acids and glycerol. As a result excess of subcutaneous fat bulges into the dermis and forms a characteristic view, typical for cellulite. Many different hormones are involved in the lipolysis process. Adrenaline, noradrenaline, glucagon and adrenocorticotropin activate the lipases, while insulin inhibits the activity of these enzymes and stimulates the collection of fat in adipose tissue [72]. Different compounds, which activate the lipolysis pathway, stimulate structural changes of the G-protein coupled receptor and stimulate the cytosolic cAMP production. Its increasing level stimulates protein kinase A, which activates hormone-sensitive lipase (HSL) by phosphorylation. The latter enzyme hydrolyses triglycerides and its activity is cAMP-dependent [7,73].

Tea, due to the content of alkaloids, is widely used in the production of cosmetics against cellulite. The major one—caffeine (known also as theine) stimulates microcirculation in the skin, which in turn improves cell oxygenation and accelerates fat burning in skin cells. Therefore, cosmetic preparations containing alcoholic or glycolic tea extracts are used to maintain a slim figure, reduce cellulite and remove toxic products from the body. Not only alkaloids, but also polyphenols, are very effective in reducing cellulite. Catechins, which are a dominant compounds present in tea extracts, were described to inhibit glycation and oxidation of proteins and thus preventing the formation of cellulite, which is one of the symptoms of skin aging. Therefore, tea is a common ingredient in cosmetic preparations with firming, slimming and anti-cellulite properties [74,75,76].

The main active ingredient of the tea is caffeine, which is frequently used as an anti-cellulite agent. It can be used as a pure compound or green tea extract ingredient. Caffeine could influence the mechanisms of cellulite formation in different ways. The most important is the acceleration of lipolysis through the influence on the catecholamine secretion and thus increasing the cAMP synthesis in adipocytes and activating HSL. This inhibits fat accumulation by increased degradation of triglycerides and therefore reduction of cellulite [77,78].

Pires-de-Campos and co-investigators studied the effect of topical gel application with caffeine using three different models: gel with ultrasound treatment (3 MHz, intensity: 0.2 W/cm^2^, rate: 1 min/cm^2^), gel with caffeine (5%, water-in-water) and gel with caffeine and ultrasound. The study was conducted using a swine hypodermis and gel was applied daily during 15 days. A specified area without any application was used as control. The study revealed that the model using caffeine and ultrasounds was the most effective. The thickness of the subcutaneous adipose tissue, damage of the adipocytes and the numbers of cells were significantly reduced [79]. Velasco and co-investigators also investigated the effectiveness of the topical application (for 21 days) of emulsion containing caffeine on the diameter and number of fatty cells using Wistar female mice. The study revealed that emulsion-containing caffeine reduced by 17% the diameter of the fatty cells [80]. Recently Byun and co-investigators evaluated the effectiveness of the slimming cream containing 3.5% of water-soluble caffeine and xanthenes for the treatment of cellulite. Fifteen subjects with cellulite applied slimming cream to the thighs and inner side of the upper arms twice daily for 6 weeks. The effectiveness was evaluated using a standard visual scale, circuit changes of the thighs and upper arms, and patient satisfaction using a questionnaire. Performed clinical study revealed significant improvement in skin condition and cellulite reduction (by 19.8% in the standard visual scale score). Thigh and upper arm circumferences decreased by 0.7 cm (1.7%) and 0.8 cm (2.3%), respectively, at week 6 and no serious side effects were observed [81] (Table 2).

All the above-mentioned experimental data confirm that caffeine, which is the major alkaloid present in tea leaves, is a very effective natural agent in the reduction of cellulite. When topically applied, it inhibits fat accumulation in the hypodermis and decreases the number of adipocyte cells. Therefore cosmetic formulations containing tea extracts or caffeine alone may be very efficient products in reducing skin cellulite and improving body figure.

Not only caffeine, but also tea polyphenols may be very effective in reducing cellulite and “slim” the figure. Weight reducing properties of different polyphenols, including tea catechins were recently described in numerous scientific papers [5]. Oral ingestion as well as topical application of tea extracts was described to reduce weight and decrease adipose tissue content. More than 24 clinical placebo-controlled trials, performed on obese patients, revealed that consumption of 600–900 mg of tea polyphenols per day (which is equal to 3–4 cups of green tea) significantly decreased total abdominal fat, reduced serum triglycerides, induced adipogenesis, increased energy expenditure and fat oxidation and improve fecal lipid excretion. As a result significant weight loss and reduction of skin fat tissue was observed [82,83,84]. In vivo studies also performed in various animal models showed that increased intake of tea extracts or its active constituents significantly decreased hepatic lipid accumulation, white adipose tissue weight and downregulated over 100 genes related to tissue inflammatory responses [85,86].

Rao and co-investigators tested a cream containing black pepper, sweet orange peel, ginger root extract, cinnamon bark extract, capsaicin, green tea and caffeine. The formulation was applied under occlusion with neoprene shorts to 20 patients. After testing 76% of the volunteers noticed an overall improvement of their cellulite, with 54% reporting greater improvement in the thigh that received garment occlusion. Professional dermatological examination revealed significant improvement of the skin of the thighs condition (average circumference reduction was 1.2 cm; 1.3 cm in the group with occlusion and a 1.1 cm reduction without occlusion) [87]. Although there are many cosmetic preparations for the treatment of cellulite, which contain different tea extracts, the literature review showed almost no studies evaluating the effectiveness of tea polyphenols in the treatment of cellulite. Their efficacy towards cellulite reduction is mostly based on their properties to treat obesity, decrease weight, reduce waist circumference and improve thermogenesis [88,89]. Therefore, there is a strong need to perform studies, which will evaluate the influence of tea polyphenols on cellulite, both orally and especially topically.

Although tea polyphenols were not studied towards their anti-cellulite activity, there are experiments that proved that products rich in polyphenols might effectively reduce cellulite. Savikin and co-investigators revealed that dietary supplementation with chokeberry juice rich in polyphenols improve skin morphology in cellulite. They examined twenty-nine women aged 25–48 with a cellulite grade 2 according to the Nurnberger–Muller scale. All patients consumed 100 mL of the juice for 90 days. Skin structure was analyzed by ultrasonography at 0, 45 and 90 days of the study. The study revealed significant reduction in the subcutaneous tissue thickness (1.9 mm on average). Moreover the length of subcutaneous tissue fascicles was reduced in 97% of subjects. It was also observed that in the subjects with edema at the baseline, after 45 days of treatment, it was reduced in 55.2% of patients, while after 90 days edemas were not observed in any of the individuals involved in the study. The authors did not investigate polyphenolic composition of the juices, they only performed determinations using a Folin-Ciocalteu reagent. Therefore it is difficult to state which particular polyphenols were primarily responsible for such an effect [90].

### 3.5. Improvement of Skin Condition

Green tea is the type of tea that is the most extensively used in cosmetic preparations improving skin and hair condition. Although an easy search for a term “green tea skin care products” allows us to find over 50 different products that can be used on skin, nails and hair. These products are targeted towards the improvement of aging skin, treating rosacea, acne, warts and increased sebum production. However after closer evaluation of the product information, it occurs that there is little information on the extract preparation, its concentration and other active ingredient content. It is generally accepted that 5% is an effective concentration of green tea extract in cosmetic formulation, however it should be remembered that catechins, being one of the most effective antioxidants, are very sensitive to light and oxygen. Therefore they require a very careful formulation to retain their biological activity. Another question is the skin permeability of catechins as these are a typical hydrophilic compounds and they penetration in human skin is limited. Thus, according to Farris, there should be a healthy dose of skepticism, for both consumers and dermatologists, regarding the effectiveness and usefulness of cosmetics containing green tea [91]. There is also little scientific data, especially clinical experiments, which support the wide use of tea extracts in cosmetics improving the condition of the skin and hair. Another question is the fact that the term “improving” is very relative and consumers may often have a big problem in proper evaluation of the cosmetic effectiveness, and their assessment may be incorrect. Only typical dermatological examination can be an appropriate tool to evaluate the efficacy of the cosmetic formulation. 

A clinical study conducted by Syed and co-workers revealed an improvement in skin texture and appearance in 45% of volunteers after a 4-week application of gel containing EGCG [92]. In another clinical study different cosmetic formulations containing vehicles supplemented with 6% *Camellia sinensis* glycolic leaf extracts were applied to the forearm skin of 24 volunteers. Before the study and after 2 h, 15 and 30 days the following parameters were evaluated: stratum corneum water content, transepidermal water loss, skin viscoelastic-to-elastic ratio (Uv/Ue) and microrelief. Obtained results revealed significant increase in skin moisture (immediate and long-term), improved skin microrelief (skin texture), represented by reduced skin roughness and enhanced skin smoothness (especially after 15–30 days of treatment). It was also proved that formulations containing green tea extract after 30 days of topical administration significantly enhanced Uv/Ue parameter in comparison to vehicle and control. The authors concluded that green tea might be a promising botanical component of cosmetics, which especially improve moisture and microrelief of the skin [42]. 

A clinical double-blind placebo study, sponsored by NuSkin (Provo, Utah, USA) was conducted to evaluate the efficacy of the combined oral supplementation of the green tea extract and topical application of the special cream containing 10% of green tea extract to improve the appearance of the photoaged skin [93]. The study involved 40 women with moderate photoaging. Eighteen individuals took a dietary supplement containing 300 mg of green tea extract (twice daily) and applied two times per day a portion of green tea cream. In the placebo group 18 women took placebo supplement and placebo cream also twice per day. All the subjects used the same sunscreen and cleanser. The study was conducted for 8 weeks. After finishing, professional dermatological external examination revealed no statistical changes of the skin of the patients from both groups. However, the skin biopsy shown significant improvement in elastic tissue content in the green tea treated group. Unfortunately, the design of the study, did not allow determining of whether the obtained effect was due to the oral intake of green tea or its topical application.

Green tea based cosmetic formulations are also popular to reduce increased sebum production, which is a main feature of an oily face. Sebum is a mixture of lipids, mainly squalene and wax esters, which is produced by sebaceous glands, especially on the face and scalp. Sebum production is hormonally regulated, and its increased production causes serious skin disorders, such as acne vulgaris [6]. Topical application of green tea extract may be very beneficial in reducing excessive sebum production and two independent clinical studies proved its effectiveness. Meethama and co-workers in a randomized single-blind, placebo-controlled study proved anti-sebum efficacy of a facial tonners containing green tea. The developed cosmetics contained 2%, 4.5% and 7% of green tea extract with 100 mg% of polyphenols, whereas the base consisted of hydroxyethyl cellulose, glycerin and panthenol. The study involved twenty healthy Thai volunteers (sixteen female and four male) aged between 20 and 35 years old. All tested products were stable and caused no skin irritation, which was proved using patch tests. Performed clinical investigation revealed significant anti-greasy and anti-sebum activity of a green tea extract, which in both cases were positively correlated with the concentration of the extract. Moreover it was proved that the effectiveness of a 28 days treatment was significantly better than 14 days. It was stated that cosmetics containing green tea extract might effectively improve the condition of oily face [94]. In a single-blinded, placebo controlled monocentric study performed by Mahmood and co-workers, a group of twenty two non-smoker, healthy men was investigated towards the effectiveness of a lotus and green tea topical application on facial sebum production. Volunteers were divided into two groups—in a first group men used green tea (5%) topical on one cheek and placebo control on another (*n* = 11). The second group used a combination of green tea and lotus (2.5% each) on one cheek and placebo control on another (*n* = 11). Both groups were asked to apply their respective topicals at bedtime for 60 days. Sebum secretion was measured using sebumeter in both groups at baseline and after 15, 30, 45 and 60 days. The study revealed a significant reduction of sebum production in both groups after 60 days of treatment—in the group applying only green tea and the combination of green tea and lotus, the sebum production was reduced by 27% and 25%, respectively. The performed study revealed that cosmetic therapy using green tea extract alone as well as a combination of green tea and lotus extracts may be a very effective tool in the treatment of skin disorders associated with increased sebum production, such as acne vulgaris [95]. In another study performed by Mahmood and co-workers ten healthy men aged 24–40 years old used topically to their cheeks a cosmetic formulation containing 3% of green tea extract. The experiment was conducted for eight weeks and a sebumeter was used to evaluate the reduction in sebum production, which was then calculated to a percentage value. Obtained results revealed significant decrease in sebum production during the eight weeks study. The strongest effect was observed after eight weeks of treatment (the reduction of 60%). However, already after the first week significant improvement was noted (10%) [96]. It should be strongly emphasized that both of the above mentioned studies had significant limitations. A small sample size, the investigated group that involved only men and no comparison treatment or placebo control (in the case of the latter study [97]) were the biggest disadvantages of both of these studies (Table 2).

### 3.6. Improvement of Hair Condition

Cosmetic preparations containing tea extracts are recommended for patients with androgenetic alopecia and hair loss, regardless of gender. The occurrence of androgenic alopecia is directly related to the conversion of testosterone into more active dihydrotestosterone (DHT), which is mainly responsible for baldness. Hair follicles are particularly sensitive to DHT, which shortens the anagen phase of the hair growth cycle. As a result, most of the hair passes into the telogen phase, which is characterized by follicle miniaturization and reduction of hair roots. The newly growing hair is weaker—thinner and shorter and after several cycles, they stop to growth and hair loss can be observed [68]. In several studies tea polyphenols, essential oils and caffeine present in tea plant leaves inhibit the activity of 5α-reductase, which results in a decreased DHT formation [96,98]. The former compounds were also found stimulate hair roots and extend the hair growth phase (anagen phase) [76]. Therefore, constituents of tea are important ingredients of hair and scalp care cosmetics, which are especially recommended to individuals having excessive greasy hair and dandruff [11,74,76].

Fischer and co-investigators performed an in vitro study, which have shown that external application of caffeine in a concentration of 0.001% and 0.005% led to a significant stimulation of human hair follicle growth. It was concluded that caffeine reduces smooth muscle tension near the hair follicle and therefore significantly increases delivery of nutrients through the microcirculation of the papillae of the hair [98].

Green tea polyphenols were proved to significantly improve hair loss in mice. A group of 30 mice were fed with 50% fraction of polyphenol extract from dehydrated green tea in their drinking water for six months, whereas the control group received regular drinking water. Both groups received the same diet. The study revealed significant improvement in hair growth (33% of animals) in comparison to control group [99]. One of the main catechins present in green tea extract—EGCG was proved to be strong 5α-reductase and aromatase inhibitor [100,101]. This fact may significantly explain the effectiveness of using green tea polyphenols to treat androgenic alopecia, which is mainly associated with increased activity of these both enzymes. Such a mechanism was proved by Kwon and co-workers who evaluated the efficacy of EGCG on human hair growth. The study revealed that EGCG stimulated hair growth in hair follicles ex vivo culture and the proliferation of cultured human dermal papilla cells. Moreover, it was shown that epigallocatechin-3-gallate promoted hair growth in vivo dermal papillae of human scalps. It was concluded that EGCG stimulates hair growth through dual proliferative and anti-apoptotic effect [102].

All the above mentioned experiments proved practical usefulness of tea extract in formulating cosmetics, which improve skin appearance and hair condition and growth. However, majority of these studies deal with green tea extract, therefore more research regarding black or white tea application should be performed.

### 3.7. Improvement of Skin Microcirculation

It is widely known that tea polyphenols improve microvessel system and microcirculation in the skin and—through different receptors—increase microvessels elasticity [37]. Polyphenols are thromboxane A2 synthesis inhibitors, which induce the anticoagulant effect. Due to their strong antioxidant properties these compounds also have a protective activity towards prostacyclines. Their influence on microvessels permeability is also indirect as they inhibit the oxidation of vitamin C, which is a key factor in collagen synthesis—a protein that is a crucial component of vascular wall [38]. There are studies, which based on the above mentioned mechanisms, proved that internal and external use of tea polyphenols may significantly improve skin microcirculation [5]. 

Heinrich and co-investigators performed a 12-week, double-blind, placebo-controlled study, involving 60 female volunteers, who were randomized to an intervention or control group. The first group received a beverage containing green tea polyphenols providing 1402 mg of catechins per day. The second group received a control beverage. Several skin parameters (skin photoprotection, structure and function) were measured at baseline, after 6 weeks and after 12 weeks. The study revealed significant improvement in skin deterioration following UV-radiation, elasticity, roughness, scaling, density and water homeostasis. What is important, the consumption of beverage rich in green tea catechins significantly increased blood flow (40% by week 6 and 29% by week 12) and oxygen delivery to the skin (from 30% at the baseline up to 38% and 40% by week 6 and 12, respectively). Within the same experiment a randomized, double-blind, single-dose (0.5, 1.0 and 2.0 g) study of green tea polyphenols, which were administrated in a form of a capsule was performed on a group of 15 female volunteers who had not participated in the 12-week study. This short-term study showed that blood flow was maximized at 30 min after ingestion. In general, performed clinical investigation proved that increased intake of green tea catechins significantly improves skin condition, including blood flow and microcirculation in the skin tissue 5]. Rothenberger and co-workers investigated whether tea tree oil increases skin blood flow, which is a very important task in wound treatment. Therefore the authors analyzed the direct effect of topical antiseptic agents, including oil from a tea tree, on the microcirculation of intact human skin. The study was conducted in a group of 20 volunteers (nine men and 11 women). Patients immersed their fore, middle, ring and little fingers of the right hand in cups with appropriate solutions, including 5% of tea tree oil, which was dissolved in 0.9% of NaCl. An oxygen to see diagnostic device was used to evaluate oxygen supply in microcirculation of blood perfused tissues (blood flow, hemoglobin oxygenation and the relative amount of hemoglobin). Obtained results showed that tea tree oil significantly increased (+19%) blood flow compared to control. Moreover, the improvement after using tea tree oil was the highest in comparison to other substances used in the study (ocetnidine and polyhexanide). The alterations in the hemoglobin oxygenation were not significant. Tea was proved to be a very effective antibacterial agent, which significantly improves skin perfusion, which is an important factor in wound healing [103] (Table 2).

Caffeine was also proved to improve the microcirculation in the skin of the head and therefore to increase nutrients delivery to the hair bulbs, which strengthens and stimulates rapid growth of the hair [104]. This alkaloid was also proved to improve the circulation in other tissues. An in vivo study performed using positron emission tomography (PET) revealed that oral administration of 250 mg of caffeine improved blood circulation in the human brain by 30% [105], whereas a dose of 100 mg given orally increased microcirculation in the human ocular fundus, which was revealed in a study performed on 10 healthy volunteers using a laser speckle tissue circulation analyzer [106]. Lupi and co-workers performed a clinical investigation in a group of 134 women with cellulite, aged between 20 and 39 years, to evaluate the effectiveness of a commercially available cosmetic (Elancyl^®^ Chrono-Active) composed mainly of a 7% caffeine solution, to improve microcirculation and reduce cellulite. Microcirculatory parameters evaluated were functional capillary density (FCD; number of flowing capillaries per unit area), diameter of the dermic papilla (DPD) and capillary diameter (CD). Moreover different clinical parameters, such as centimetrical measurements of thighs and hips, were evaluated. Additionally the influence of tobacco smoking, alcohol consumption and physical activity on the efficacy of the treatment was assessed. The study was conducted for 1 month. Obtained results revealed an increase of the all microcirculation parameters (FCD, DPD and CD) in the studied group. However, the changes were not statistically significant. Thigh and hip circumferences were significantly reduced, in more than 80% and 67% of cases, respectively. Moreover, it was shown that tobacco smoking, alcohol consumption and the level of physical activity had no influence on the circumference of the treated thighs and hips [107]. Table 2 summarizes studies and their results, which were performed regarding the beneficial effects of tea and its active constituents towards the skin.

## 4. Skin Penetration of Tea Active Constituents

The efficacy of cosmetic formulation is strictly correlated with the skin permeability of its active ingredients. One of the most important penetration-conditioning factors is the polarity of components. According to Yanagida and co-investigators [108] the partition coefficients (K(non-polar/polar)) of green tea polyphenols can be placed in the following order: ECG (6.25) > EGCG (2.94) > EC(2.38) > C(2.33) > EGC(0.93). From all simple catechins present in tea extract EGC is considered as the most hydrophilic molecule, while ECG is characterized by the smallest polarity [108]. A study performed by Dal Belo and co-investigators [69] revealed that, after topical application of a green tea extract containing cream, EGCG was significantly retained within the skin, mostly in the stratum corneum, followed by the epidermis and dermis. This suggests a non-polar character of EGCG, as non-polar compounds tend to stay within the stratum corneum layer, and not penetrate into deeper parts of the skin, like the epidermis and dermis [109]. This is in agreement with a previous finding of Yanagida and co-workers, which also suggests a non-polar character of EGCG [108]. Zillich and co-investigators reported that both the size of the molecule as well as the hydrophobicity are the most crucial parameters regarding the ability of green tea catechins to penetrate the skin [6]. Definitely more experiments, especially in vivo and human studies, are needed to expand the knowledge on the bioavailability of tea polyphenols from cosmetic preparations.

Caffeine, a very important tea active constituent, is more often used as a hydrophilic model substance in skin penetration experiments. An in vitro study performed by Van de Sandt and co-workers [110] revealed that the maximal absorption rates of caffeine through the human skin were found to be 2.24 ± 1.43 µg/cm^2^/h. The authors have also proved that the maximal absorption of this alkaloid was reached 100 min after percutaneous application in the human skin [111]. Touitou and co-investigators studied caffeine skin delivery by carrier design [112]. Using quantitative skin autoradiography they have confirmed the highest concentration of caffeine (280 µg/g tissue) after 24 h in the epidermis, while the lowest amount of this alkaloid (50 g/g tissue) was detected in the dermis. However, the caffeine’s ability to penetrate from different cosmetic preparations through the skin barrier differs and is significantly correlated with the type of emulsion applied on the skin [72]. It occurs that its permeation mostly depends on the quantity of the formulation applied, rather than on the concentration of caffeine in the cosmetic formulation [113]. It was also proved that the composition of the cosmetic significantly affects caffeine absorption through the skin—water-in-oil nanoemulsion formulations were much more effective in comparison to aqueous solutions of caffeine, which was proved in Franz diffusion cells using rat skin as permeation membrane [114]. An effective way to transfer caffeine through the skin barrier was based on the application of microspheres in aqueous suspension (diameter of the microspheres: 2.8 µm, caffeine loading: 2.3 mg/g of particles). Such a formulation improved caffeine transfer across the skin, as microspheres easily penetrated the skin barrier and gathered in the receptor compartment, providing continuous alkaloid release [71,115]. Taking all into consideration it should be remembered that not only the quality of the tea extract or the concentration of caffeine, but first of all the composition of the cosmetic formulation is significant for the effectiveness of topical applications containing this alkaloid. 

## 5. Skincare Products Containing Tea Extracts

Tea extracts are important components of many cosmetics, including creams, moisturizing lotions, tonics, shower gels, hair products as well as cosmetic facial masks. That frequent use of *Camellia sinensis* extracts is due to its multidirectional effect. From all types of tea extracts, those obtained from green tea are the most widely used. These are proposed not only for young and problematic skin types, as they inhibit excessive sebum production, but can also be used by people with sensitive and allergic skin [11,38]. However, on the market also cosmetic products containing black and white tea can be found and their popularity is increasing. Due to a many producers and cosmetic forms a large number of different products can be found and their exact number is hard to estimate, also because almost every year new products appear on the market. Table 3 presents an example of 30 different cosmetics containing green, black and white extracts, including a type of a cosmetic form and manufacturer′s recommendations. 

According to Możdżeń and co-workers, who analyzed the plant species, which are used to produce cosmetic masks offered on the Polish market, *Camellia sinensis* is the most commonly used, followed by *Matricaria chamomilla* L. and *Vitis vinifera* L. [116].

After applying cosmetics containing tea extract, the skin appears more tense and refreshed, which is the result of astringent activity of polyphenols and tannins and their interaction with keratin present in the stratum corneum. This process also leads to a reduction in skin redness, irritation and reduction of swelling. The facial masks, containing mainly green tea extracts, have a disinfecting, antioxidant and toning effect. They also soothe inflammation, accelerate the healing of wounds and skin eruptions, and also close skin pores by which they reduce their visibility [116]. 

Tea infusions are also used as compresses, which are applied after insect bites [76] or to soothe the itching and burning around the eyes. 

The latter effect results from the vasoconstriction induced by the metabolites of tea plant and is shown up as a noticeable reduction of swelling around the eyes, which is often observed during inflammation [11,76]. 

## 6. Conclusions and Perspectives

Plentiful of biological effects of *Camellia sinensis* presented in this review shed light on the application of its extracts in cosmetics apart from its better known usage in pharmaceutics. Tea plant leaves are the most rich sources of catechins among plants, but also deliver caffeine—a purine alkaloid of high cosmetic significance. Together with the development of the analytical instrumentation and biological activity evaluation methodology, further precious properties of this plant were revealed towards skin. The administration of tea plant in cosmetics seems to be interesting and precious, due to the fact that it does not have any confirmed allergenic or irritating effects after topical administration. Low toxicity of single metabolites of its extracts, often appearing as synergistic actions with other antioxidants commonly added to different types of products and additional preserving properties towards final cosmetics formulations encourages the use of tea plant metabolites in various applications. 

Among the benefits of tea plant and its extracts, its value in the anti-ageing treatment, skin and hair care and slimming properties should be underlined. Its constituents are also effective in the skin microcirculation enhancement and photoprotection against harmful effects of UV irradiation.

Therefore, it is of the highest importance to collect funds on the *Camellia sinensis* fermentation type-bioactivity studies to reveal some more relations between the type f manufacturing process and the obtained biological effects. Still the largest quantity of scientific results has been obtained for the green tea extracts. The other types of leaves’ fermentation due to their different composition needs further investigations, as they are also responsible for noticeable effects towards skin. On the other hand, further trials on human skin or humans of all types of tea are necessary to confirm its therapeutic benefits already revealed in in vitro models.

Certainly, this still insufficiently studied plant in terms of its cosmetic applications will draw attention of researchers working in this sector in the nearest future.

## Figures and Tables

**Table 1:** The antioxidant activity assessment of some plant extracts used in cosmetics (TPC—total phenolic content, Folin—Ciocalteu method, TE—trolox equivalents, GAE—gallic acid equivalents).

No	Type of Extract	Antioxidant Test Applied	Antioxidant Properties	References
1	Black tea	TPC	58.2 mg/100 mL	[21]
1	Black tea	ABTS (TE)	3.11 mM/L	[21]
2	Green tea	TPC	97.3 mg/100 mL	[50]
3	Green tea	ABTS (TE)	12.6 mM/L	[50]
4	Hawthorn	DPPH	IC_50_ = 48 μg/ml	[51]
5	Chokeberry	TPC (GAE)	0.957 mg/mL	[52]
6	Grapevine (red wine)	TPC	294.41 mg/100 mL	[53]
7	Aloe	ABTS (IC50)	0.105 mg/Ml	[54]
	gel		0.033 mg/mL	
	leaf		0.30 mg/mL	
	flower			
8	Horse chestnut	TPC (GAE)	2.12 mg/g	[55]

**Table 2:** Beneficial effects of tea and its active constituents towards the skin-summary.

Activity	Experimental Model		Type of Tea/Active Constituent	Mechanism of Action/Effect	Reference
	In vitro	In vivo/Ex vivo			
Antioxidant	DPPH, ABTS, FRAP assays	Different animal models	Tea catechins (EC, EGCG, EGCG)	• Direct ROS and RNS scavenging • Chelation of trace elements, which are involved in free radicals generation (Cu, Fe) • Increasing of the endogenic antioxidant enzymes production (SOD, glutathione) • Inhibition of enzymes involved in ROS generation (glutathione S-transferase, microsomal monooxygenase, mitochondrial succinoxidase or NADH oxidase) • Protection and regeneration of antioxidant substances (vitamin C or E)	[5,43,44,47,48]
		Phase II, clinical evaluation	Topical application of gel containing EGCG	Improvement in skin texture and skin appearance in 45% of volunteers after a 4-week application	[92]
		Double-blinded, placebo-controlled trial 40 women with moderate photoaging 8 weeks of treatment	Green tea extract - oral supplementation (300 mg, 2 per day) - topical application of 10% green tea cream	- Skin biopsies revealed significant improvement in the elastic tissue content - No clinically significant changes regarding protection from cutaneous signs of photoaging were detected	[93]
		24 volunteers	Different cosmetic formulations containing vehicles supplemented with 6% Camellia sinensis glycolic leaf extracts Application to the forearm skin	- Significant increase in skin moisture (immediate and long-term) - Improved skin microrelief (skin texture) (especially after 15–30 days of treatment) - Significant enhancement of the skin viscoelastic-to-elastic ratio (Uv/Ue; after 30 days)	[41]
Photoprotective	Normal fetal lung fibroblasts (MRC5) Adult skin fibroblasts (84BR) Normal human epidermal keratinocytes (NHEK)	Peripheral blood cells obtained from10 healthy non-smoking volunteers (six female and four men)	EGCG (in vitro assay) 540 mL of green tea infusion (in vivo study)	- Significant inhibition of UVR-induced damage to peripheral blood cells - Dose-dependent inhibition of the UVR-induced DNA damage in fibroblasts and keratinocytes cell cultures - 250 µM of EGCG was found to be optimal concentration to inhibit DNA cell damage caused by UVR	[63]
		21 voluntary healthy participants Skin patches irradiated with UVB (up to 100 mJ/m^2^)	External application of a water in oil emulsion containing 4% of green tea extract	- Significant decrease of UV-induced p53 expression in keratinocytes - Significant reduction of apoptotic keratinocytes - Significant reduction of the UV-induced erythema and thymidine dimer formation	[72]
		A double-blind placebo-controlled assay - 10 healthy volunteers - skin explants (ex vivo study)	White and green tea extracts applied topically in a specially prepared cosmetic vehiculum	- Significant reduction of cutaneous immunity in UV-irradiated skin (stronger in the case of green tea) - Prevention of UV-induced oxidative DNA damage in the form of 8-OHdG (8-hydroxy-2′-deoxyguanosine)	[66]
		24 male albino hairless mice (HRS/J-hairless, Jackson, Bar Harbor, ME, USA)	Green tea and Ginkgo biloba glycolic extracts Topical formulation containing 6% of each extract applied in two different areas in a concentration of 5 mg/cm², 15 min prior UV irradiation	- Significant reduction of the UV-induced skin damage (dryness, irritation, presence of erythema, sunburn cell formation and epidermal hyperplasia) - Much stronger effect when both extracts were applied together - Strong photoprotective activity due to biological effects caused in the skin and not photo-absorption properties of both herbal extracts	[69]
Anticellulite and slimming properties		More than 24 clinical placebo-controlled trials performed on obese patients	Oral ingestion of 600–900 mg of tea polyphenols per day (equal to 3–4 cups of green tea)	- Significant reduction of the total abdominal fat - Significant reduction of the weight - Significant reduction of the skin fat tissue - Reduced serum triglycerides - Induced adipogenesis - Increased energy expenditure–increased fat oxidation - Improved fecal lipid excretion	[5,82,83,84]
		Swine hypodermis	Caffeine Three models: - Gel with ultrasound treatment (3 MHz, intensity: 0.2 W/cm^2^, rate: 1 min/cm^2^), - Gel with caffeine (5%, water-in-water) - Gel with caffeine and ultrasound Application during 15 days	- Model using caffeine and ultrasound was proved to be the most effective - Significant reduction of the subcutaneous adipose tissue, damage of the adipocytes and the numbers of cells	[79]
		Wistar female mice	Cosmetic emulsion containing 4% of caffeine applied topically for 21 days	- Significant reduction (by 17%) of the fatty cells diameter	[80]
		Clinical study - 15 healthy female volunteers with cellulite on the thigh and medial side of the upper arms	Slimming cream containing 3.5% of water-soluble caffeine and xanthenes for the treatment of cellulite Topical application twice daily for 6 weeks	- Significant improvement in skin condition - Significant reduction of the cellulite (by 19.8%) - Reduction of thigh (0.7 cm-1.7%) and upper arm (0.8 cm-2.3%) circumferences - No side effects	[81]
		Double-blinded, randomized trial 20 women with a moderate degree of cellulite 4-week treatment	Anti-cellulite cream containing black pepper, sweet orange peel, ginger root extract, cinnamon bark extract, capsaicin, green tea and caffeine. Topical application Combination of anti-cellulite cream and occlusive bioceramic-coated neoprene garment	- 76% volunteers reported improvement of their cellulite - 54% women declared greater improvement in the thigh that received garment occlusion - Significant improvement of the skin of the thighs condition (average circumference reduction was 1.2 cm; 1.3 cm in the group with oclussion and a 1.1 cm reduction without occlusion)	[87]
		29 women aged 25–48 with a cellulite grade 2 according to the Nurnberger–Muller scale	Dietary supplementation with chokeberry juice rich in polyphenols 100 mL/day for 90 days	- Significant reduction in the subcutaneous tissue thickness (1.9 mm on average) - Length of subcutaneous tissue fascicles was reduced in 97% of subjects - Reduction of edema in 55.2% of patients after 45 days of treatment - Lack of edema in all subjects after 90 days of treatment - No qualitative or quantitative determinations of the phenolic composition of the juices were performed	[90]
Improvement of skin and hair condition		Clinical double-blind placebo study 36 participants (18 in the study group and 18 in the placebo group)	Dietary supplement containing 300 mg of green tea extract (twice daily) and topical application of a green tea cream (10%)—two times per day, for 8 weeks	- No statistical changes of the skin of the patients from both groups (dermatological examination) - Skin biopsy revealed significant improvement in elastic tissue content in the green tea treated group	[93]
		Randomized single-blind, placebo-controlled study 20 healthy Thai volunteers (16 female and 4 male) aged between 20 and 35 years old	Cosmetics preparation, which contained 2%, 4.5% and 7% of green tea extract with 100 mg% of polyphenols (the base consisted of hydroxyethyl cellulose, glycerin and panthenol). Topical application for 28 days.	- Significant anti-greasy and anti-sebum activity of a green tea extract - High efficiency in the improvement of oil face condition - The activity was highly correlated with the concentration of green tea extract - The effectiveness of a 28 days treatment was significantly better than 14 days - Patch testes revealed no irritation properties	[94]
		Single-blinded, placebo controlled monocentric study 22 non-smoker, healthy men	Cosmetic preparation containing lotus and green tea extract. Application on cheek at bedtime for 60 days. Sebum production was evaluated at baseline and after 15, 30, 45 and 60 days.	- Significant reduction of sebum production - Higher efficacy in the group applying only green tea, compared to a combination of green tea and lotus (not statistically significant) - The highest effectiveness was achieved after 60 days of treatment	[95]
		Clinical investigation-10 healthy men aged 24–40 years	Cosmetic formulation containing 3% of green tea extract applied to the cheeks for 8 weeks.	- Significant decrease in sebum production - The highest efficiency was achieved after 8 weeks of treatment (60% of sebum reduction) - Significant improvement already after 1 week of treatment (sebum production decreased by 10%)	[97]
	Hair follicles from 14 biopsies, taken from the vertex areas from male with androgenic alopecia		Caffeine External application of caffeine in a concentration of 0.001 and 0.005%	- Significant stimulation of hair follicle growth - Reduction of a smooth muscle tension near the hair follicle - Significant increase of the nutrients delivery to a hair papillae	[98]
		Placebo controlled study 30 mice	Animals were fed with a diet enriched with 50% fraction of polyphenol extract from dehydrated green tea in their drinking water for six months	- Significant improvement in hair growth (33% of animals) in comparison to control group	[99]
	Cultured human dermal papilla cells	Hair follicles ex vivo culture Three normal human volunteers	EGCG 0.01, 0.1 or 0.5 μM (in vitro) 0.1, 1 or 5 μM (ex vivo) 10% in ethanol or ethanol vehicle (in vivo)	- Stimulation of the culture cells growth - Proliferative and anti-apoptotic effect towards dermal papillae of human scalps - Prolongation of anagen stage	[102]
Improvement of skin microcirculation		Double-blind, placebo-controlled study 60 female volunteers	Green tea beverage (1402 mg of catechins per day) for 12 weeks.	- Significant improvement in blood flow (40% by week 6 and 29% by week 12) - Significant improvement in oxygen delivery to the skin (from 30% at the baseline up to 38% and 40% by week 6 and 12, respectively) - Significant improvement of several skin parameters (elasticity, roughness, scaling, density and water homeostasis)	[5]
		Randomized, double-blind, single-dose study 15 female volunteers	Green tea extract (0.5, 1.0 and 2.0 g) administrated orally in a form of capsule	- Maximized blood flow 30 min. after ingestion - Significant improvement of skin condition - Increased blood flow and microcirculation in the skin tissue	[5]
		Clinical investigation in a group of 20 volunteers (nine men and 11 women) with wounds	Topical application of antiseptic agent containing tee tree oil (5% in a saline)	- Significant increase (+19%) of blood flow compared to control - Significant improvement of wound healing (in comparison to ocetnidine and polyhexanide) - Alterations in the hemoglobin oxygenation (not significant) - Significant improvement of skin perfusion - Strong antibacterial activity	[103]
		Clinical investigation in a group of 134 women with cellulite, aged between 20–39 years	Commercially available cosmetic (Elancyl^®^ Chrono-Active) containing 7% of caffeine Topical application for 1 month	- Insignificant improvement of skin microcirculation (functional capillary density, number of flowing capillaries per unit area, diameter of the dermic papilla and capillary diameter) - Significant reduction of thigh and hip circumferences in 80% and 67% of cases, respectively - No influence of alcohol consumption, tobacco smoking and level of physical activity on thigh and hip circuit was revealed	[107]

**Table 3:** Selected examples of cosmetic products containing tea.

Tea Extract	Cosmetic Product	Cosmetic’s Effects (Manufacturer′s Declaration)	Production Area
Green tea	Peeling mask	- Improved skin regeneration based on the antioxidant, anti-inflammatory and toning properties of green tea	Poland
Green tea	Face mask	- Strong soothing, anti-inflammatory and regenerative properties - antioxidant activity - Protection against harmful environmental influences	Poland
Green tea	Face mask	- Eliminated excess sebum - Proper skin hydration	USA
Green tea	Shampoo	- Hair care for normal and slightly damaged hair - Antioxidant properties - Soothing action towards sensitive scalp (slightly moisturized and refreshed)	Poland
Green tea (Fuji)	Shampoo	- Hair care for normal hair - Refreshed and purified hair and scalp	UK
Green tea (Matcha)	Shampoo	- Reduction of dandruff and greasy hair - Clarified and toned scalp - Intensive shine	UK
Green tea	Shampoo	- Antioxidant in hair care (protection of hair against free radicals) - Hydrated and moisturized scalp	Malaysia
Green tea	Hair conditioner	- Hair care for all hair types - Protection against moisture loss - Strong antioxidant and hair growth stimulant properties - Smooth and soft hair	UK
Green tea	Hair conditioner	- Strengthens hair - Antioxidant properties towards hair	Japan
Green tea	Hand and Body Lotion	- Nourishing cream for feet, hands and body - Makes the skin smooth and hydrated	USA
Green tea	Body lotion	- A fresh fragrance - Refreshing body and mind - Improved mood	USA
Green tea	Balancing lotion	- Superior hydration and nourishment of the skin - Softened and smoothed, cleansed skin leaving	USA
Green tea	Refreshing body lotion	- Hydrated skin - Skin fragrance for a long time	USA
Green tea	Body cream	- Nourished and moisturized skin - Smoothed skin - Soft and flexible skin - Skin care for all skin types	USA
Green tea (Fuji)	Hand cream	- Nourished hands’ skin - Softer and smoother hands	UK
Green tea (Matcha)	Hand cream	- Skin care for all skin types - Nourished and moisturized skin of the hands	UK
Green tea	Eye cream	- Removed six types of wrinkles under the eyes - Improved production of hyaluronic acid by epidermal cells - Restored moisture in the skin to fill fine lines in dry skin	Japan
Black tea	Face mask	- Nourished and smoothed skin - Antioxidant properties towards the skin	USA
Black tea	Instant perfecting mask	Black tea complex: - Protection against harmful effects of the environment - Reduction of the skin roughness, improved glow and elasticity - Softened, smoothed and soothed the skin	EU
Black tea (Darjeeling tea)	Antiwrinkle cream	- Protection against any harmful environmental factors - Moisturized and revitalized skin	South Korea
Black tea	Shampoo	- Everyday shampoo for all types of hair - Removed build-up and excess oils	UK
Black tea	Lotion	- Hydration and the look of firm, radiant skin - Provided SPF 20 UVA/UVB sun protection - Protection against UV and free radicals-induced damage Recommended for: - Dryness - Dullness/uneven texture - Loss of firmness/elasticity	EU
Black tea	Body cream	- Antioxidant benefits towards the skin - Smoothed and moisturized the skin	USA
Black tea	Firming corset cream	Black tea complex: - Firms and redefines the contours - Prevents loss of elasticity and smoothness - Makes the skin look healthier and younger - Protects the skin against free radicals	EU
Black tea	Eye concentrate	Black tea complex: - Firmed and redefined eye contour - Increased elasticity and smoothness - Healthier and more youthful appearance	EU
Black tea	Hand cream	- Hand skin care for all skin types - Regenerated and smoothed the skin	UK
White tea	Body cream	- Softened and hydrated skin	USA
White tea	Shower gel	- Refreshed and softened fragrant skin	USA
White tea	Toilet water	- Fragrant skin	USA
White tea	Hand cream	- Alleviated rough patches and calluses	USA

## Abbreviations

C	(+)-catechin
cAMP	adenosine cyclic 3′5′-monophosphate
CD	capillary diameter
CD1a+	epidermal Langerhans cells
COX-1	cyclooxygenase 1
DHT	dihydrotestosterone
DPD	dermic papilla diameter
EC	(–)-epicatechin
ECG	(–)-epicatechin gallate
EGC	(–)-epigallocatechin
EGCG	(–)-epigallocatechin gallate
FCD	functional capillary density
GA	gallic acid
GC	(–)-gallocatechin
GCG	(–)-gallocatechin gallate
HSL	hormonesensitive lipase
IL-10	interleukin 10
NaCl	natrium chloride
NHEK	normal human epidermal keratinocytes
RNS	reactive nitrogen species
ROS	reactive oxygen species
SOD	super oxide dismutase
TB	theobrownins
TF	theaflavins
TF1	theaflavin
TF2A	theaflavin 3-gallate
TF2B	theaflavin 3′-gallate
TF3	3-3′-theaflavin digalusate
TR	thearubigins
TXAS	thromboxane synthase
UV	ultraviolet
UVR	ultraviolet radiation
